# An evaluation of the Corning 902 direct potentiometric sodium/potassium analyser

**DOI:** 10.1155/S1463924682000327

**Published:** 1982

**Authors:** P. Bijster, H. L. Vader, C. L. J. Vink

**Affiliations:** Klinisch Laboratorium Sint Joseph Ziekenhuis Aalsterweg 259 Eindhoven 5644 RC The Netherlands


Journal of Automatic Chemistry, Volume 4, Number 3 (July–September 1982), pages 125–128

### An evaluation of the Corning 902 direct potentiometric sodium/potassium analyser

**P. Bijster*, H. L. Vader and C. L. J. Vink**

*Klinisch Laboratorium, Sint Joseph Ziekenhuis, Aalsterweg 259, 5644 RC Eindhoven, The Netherlands*

### Introduction

An increasing number of clinical laboratories are using direct potentiometric analysers for the measurement of sodium and potassium in whole blood, plasma and serum. Apart from being superior to flame photometry—ionic activities are measured in the plasma water-phase rather than in 'whole plasma' [1–3]—analysis with ion-selective electrodes (ISE) in an undiluted sample can be performed with heparinized whole blood and this feature makes direct potentiometry suitable for stat analysis.

### Apparatus

The Corning 902 sodium/potassium analyser measures sodium and potassium activity in an undiluted sample by direct potentiometry. The 902's sodium electrode consists of a glass capillary, which is selective to sodium ions. The sample is passed through the capillary, with the outer surface of the glass membrane in contact with the electrode's solution. The potassium electrode consists of a valinomycin-based membrane, which is in contact with both the sample and the solution in the electrode. The reference electrode is a shell filled with 4 M potassium chloride and is separated from the sample by a cellulose membrane. The electrical connections are made via a silver/silver chloride wire.

The sample volume required is 135 $\mu$l for whole blood, plasma or serum in the 'normal mode'. To fill the electrode block only, 65 $\mu$l of sample is needed (this is called the 'micro mode').

Most of the other commercially available analysers perform a fully automatic one-point calibration after each sample measurement, to correct for possible electrode drift. The Corning 902 analyser is not automatically calibrated after each sample measurement, rather, it switches to stand-by operation 4 min. after the last measurement was performed. Further samples can then only be measured after a manual one-point calibration has been performed.

Two standard solutions are used for calibration. The 'calibration standard' is used for one-point calibration and to automatically flush the electrode block after each determination; and the 'slope standard' is used together with the calibration standard to perform two-point calibration. The analyser is designed to be connected to an a.c. supply at all times, so the calibration sequence is started from 'standby'. After the 'cal' button is pressed, the 902 will carry out an automatic one-point calibration. After a 'data ready' LED has been illuminated, a tone will indicate that the flush sequence is complete. If the 'slope' LED flashes at this point, a two-point calibration needs to be carried out—at least 8 h must have elapsed since a slope sequence was performed. Otherwise, samples can be determined.

To perform a two-point calibration the slope standard is presented manually to the sample probe of the analyser and, when the 'slope' button is pressed, the 902 draws up sufficient solution to perform a slope calibration. After the 'data ready' LED is illuminated, a tone indicates that the flush sequence is complete and that samples can be determined. Samples can be presented to the sample probe, when it is raised, using a syringe, in a cup, test-tube or capillary (using an adaptor). One operating cycle is completed within 42 s.

The output signals of the sodium and potassium channels are read approximately every 500 ms. Ten readings must be within $\pm 0{\cdot}4$ mmol/l $Na^+$ and $\pm 002$ mmol/l $K^+$ of the mean of the previous 10 readings in order to obtain a displayed concentration. To ensure short-term stability, the 10 consecutive readings must be within a range of 1 mmol/l $Na^+$ and 0·05 mmol/l $K^+$. The drift between two calibrations is also monitored: if it exceeds $\pm 2$ mmol/l $Na^+$ or $\pm 0{\cdot}06$ mmol/l $K^+$ further calibration has to be carried out before continuing with sample determinations. The system has to be cleaned weekly with a deproteinizing solution. After the cleaning procedure an electrode conditioning solution (0·1 mmol/l ammonium hydrogen difluoride) is used to activate the sodium electrode.

### Materials

Although not strictly correct, the term 'concentration' is used in this paper instead of 'activity'. ISEs respond to activity, but standards with known activity were not used in the evaluation so the results are not expressed on the activity scale. All reagents used were obtained from Corning. The calibration standard (Corning Cat. No. 00156041J) consists of 140 mmol/l of sodium chloride and 4·00 mmol/l of potassium chloride in a Tris-buffer (pH 7·4). The slope standard (Corning Cat. No. 00156042K) contains 160 mmol/l of sodium chloride and 2·00 mmol/l of potassium chloride in the same buffer. Both standards contain a surfactant.

To determine the between-day precision a serum pool and a control serum pool of several vials of reconstituted control-serum (Autonorm 216, Nyegaard and Company, PO Box 4220, Torshov, Oslo 4, Norway) were used. Aliquots of 1 ml were frozen at $-20°C$.

Sodium chloride p.a. (Merck art. 6404) and potassium chloride p.a. (Merck art. 4936) were dried at 125°C for 48 h and then stored in a desiccator with Sicapent (Merck art. 543).

Tris (hydroxymethyl)-aminomethane p.a. (Merck art. 8382) and Tris (hydroxymethyl)-aminomethane-hydrochloride A grade (Calbiochem no. 648313) were also stored in a desiccator.

Calculated quantities of the dried materials were dissolved in bidistilled water and the test solutions were made up to volume with bidistilled water.

* *All correspondence should be sent to Dr Bijster.*

0142 0453/82/0403 0125 S05·00 © 1982 Taylor & Francis Ltd

Blood was drawn in Vacutainer tubes (Becton Dickinson B.V., Postbus 59, 3800 AB Amersfoort, The Netherlands) coated with LiF.

### Methods

### *Precision*

Intra-assay precision was determined with whole blood and plasma. As the Corning 902 does not perform a one-point calibration after each sample measurement automatically, the intra-assay precision was determined by measuring, 15 times, the whole blood and plasma samples after manually performing a two-point calibration before each sample, after manually performing a one-point calibration before each sample and in a series of 15 measurements without intermediate calibration, respectively. To assess inter-assay precision, aliquots of the serum pool and the control-serum pool were measured each in duplicate over an 18-day period after a manually performed two-point calibration.

### *Linearity*

A series of seven aqueous standard solutions with sodium concentrations ranging from 100·0 mmol/l to 195·0 mmol/l and a constant potassium concentration of 5·0 mmol/l was used to check the response of the Corning 902 for sodium.

The instrument's response for potassium was checked with five aqueous standard solutions with potassium concentrations ranging from 2·0 mmol/l up to 9·5 mmol/l and a constant sodium concentration of 140·0 mmol/l. Every standard solution was measured in duplicate and each measurement was made after a one-point calibration.

### *Carry-over*

Two aqueous standard solutions, one with high sodium and potassium chloride concentrations (1000 mmol/l and 100 mmol/l, respectively) and the other with relatively low concentrations (100·0 mmol/l and 4·0 mmol/l, respectively) were consecutively introduced into the analyser. The concentrations of the low standard were determined in triplicate before and after the introduction of the high standard. The same experiment was performed with a serum pool with and without the addition of sodium chloride and potassium chloride at concentrations of 1000 mmol/l and 100 mmol/l, respectively.

### *Influence of pH*

The sodium and potassium concentrations were measured in five aqueous standard solutions with a constant sodium concentration (140·7 mmol/l), a constant potassium concentration (4·0 mmol/l), a constant TRIS concentration (50·0 mmol/l) and different pH values (ranging from 6·5 up to 10·2). The pH values illustrated in figure 1 were measured after the preparation of the standard solutions.

### *Influence of erythrocytes*

The sodium and potassium concentrations in 50 whole blood samples were measured. After centrifuging the closed tubes in a thermostatted centrifuge for 10 min. at 1000 g at 20 ± 1°C, the concentrations were measured immediately in the plasma samples. All analyses were performed in triplicate after a single-point calibration. The influence of the haematocrit on sodium concentration was investigated by separating the erythrocytes and the plasma by centrifugation and remixing different quantities of erythrocytes and plasma to obtain 'whole blood' with increasing haematocrit. The analyses were performed in quadruplicate on blood samples from three different patients.

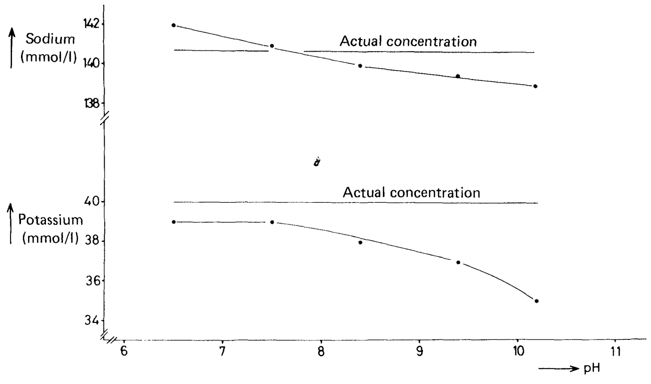

*Figure 1. Influence of pH on the displayed sodium and potassium concentration.*

The influence of haematocrit on the potassium concentration was not investigated, as Ladenson [4] has demonstrated that the potassium concentration can increase during the mixing of whole blood.

### *Influence of electrode drift after a standby period*

After a 2 h standby, a plasma pool was analysed consecutively 20 times without intermediate calibration. Before the measurement of the series a two-point calibration was performed. The sample was offered every minute. This experiment was repeated after a period of two days' standby.

### *Direct potentiometry versus flame photometry*

Serum samples from 50 patients were analysed on a flame photometer (IL 543) and on the Corning 902 in triplicate after a one-point calibration. The flame photometer was calibrated with two aqueous standard solutions (130·0 mmol/l of sodium chloride and 4·50 mmol/l of potassium chloride; and 14·0 mmol/l of sodium chloride and 4·50 mmol/l of potassium chloride, respectively). Dilution for flame photometry was carried out with a viscosity independent dilutor (Corning 800) because Vader *et al.* [4] showed that sample viscosity influences the dilution with a roller-pump.

### Results and discussion

### *Precision*

Table 1 shows the results of the intra-assay precision after a one-point calibration determined with plasma and whole blood and the results of the inter-assay precision. No significant improvement was observed on the precision following two-point calibration or without intermediate calibration. Overall, the observed intra- and inter-assay precisions for sodium and potassium are about the same as those found by Ladenson [6] and Annan *et al.* [7] with a more automated instrument.

### *Linearity*

The Corning 902 gives a linear response for sodium over the concentration range 100–195 mmol/l ($y = 1{\cdot}8 + 0{\cdot}98\,x, r = 0{\cdot}99997$; where $x$ = actual sodium concentration and $y$ = displayed sodium concentration). A slightly negative bias from the actual sodium concentration was found although this is not clinically significant. Furthermore, it is evident that the displayed potassium concentrations decrease with increasing sodium chloride concentration. This decrease is probably caused partly by an activity decrease due to increasing ion strength and partly

*Table 1. Intra-assay precision in plasma and whole blood (N = 15) and inter-assay precision in pooled human serum and control serum (N = 18).*

| | Sodium | | | | Potassium | | | |
|---|---|---|---|---|---|---|---|---|
| | Intra-assay | | Inter-assay | | Intra-assay | | Inter-assay | |
| | Plasma | Whole blood | Pooled serum | Autonorm 216 | Plasma | Whole blood | Pooled serum | Autonorm 216 |
| Mean (mmol/l) | 142·0 | 140·7 | 143·2 | 131·1 | 3·46 | 3·87 | 4·30 | 3·14 |
| SD (mmol/l) | 0·0 | 0·6 | 0·5 | 0·8 | 0·02 | 0·02 | 0·04 | 0·05 |
| CV (%) | 0·0 | 0·4 | 0·3 | 0·6 | 0·5 | 0·4 | 1·0 | 1·7 |

by the changes of the liquid junction potential of the reference electrode.

It is also clear from the results that the Corning 902 gives a linear response for potassium over the concentration range 2·0–9·5 mmol/l ($y = -0·29 + 1·05\,x$, $r = 0·99996$; where $x$ = actual potassium concentration and $y$ = displayed potassium concentration). There is a slight positive difference in the lower and a slight negative difference in the upper concentration range between actual and displayed potassium concentration. From 5·75 mmol/l up to 7·00 mmol of potassium chloride there is hardly any difference (< 0·05 mmol/l) between the concentrations. In the physiological reference area (4·0–5·0 mmol/l potassium) the displayed potassium concentration is between 0·10 and 0·06 mmol/l lower than the actual potassium concentration.

### *Carry-over*

By dividing the differences between the mean values of the low aqueous standard before and after introducing the high aqueous standard by the concentration of the high standard, a carry-over less than 0·1% both for sodium and potassium was found. The same result was found when the low serum pool was analysed before and after the introduction of the high serum pool. This proves that the wash-cycle is efficient.

### *Influence of pH*

From figure 1 it is evident that an increasing pH (and thus a decreasing hydrogen ion concentration) causes a decrease in the displayed sodium and potassium concentrations. Between pH 7·6 and 6·6 a difference of 1·0 mmol/l sodium and 0·02 mmol/l potassium was found which is similar to that found by Ladenson [5] namely 1·0 mmol/l sodium and 0·04 mmol/l potassium, respectively. Although the pH of plasma *in vitro* can increase from 7·4 up to more than 8·5, this pH increase results in a decrease of the sodium concentration which is clinically insignificant (about 0·1 mmol/l).

### *Influence of erythrocytes*

The mean difference between the sodium concentrations in 50 whole blood and plasma samples was +0·7 mmol/l (SD = 0·6 mmol/l). The same difference for potassium concentrations in whole blood and plasma was −0·02 mmol/l (SD = 0·04 mmol/l). This small difference between the concentrations in whole blood and plasma is probably caused by the influence of erythrocytes at the liquid junction potential. Due to the high potassium chloride concentration of the reference electrode solution the erythrocytes seem to haemolyse and proteins to precipitate at the liquid junction. This phenomenon has been described by Maas [8] for the pH measurement.

Instruments with a modified liquid junction, like the Corning 902, give a small difference compared with instruments with an open static liquid junction [9]. The relation between the sodium concentrations and haematocrit is shown in figure 2 for three patients. The Corning 902 displays a significantly higher sodium concentration in whole blood than in plasma at high haematocrits, this effect has also been seen with other analysers.

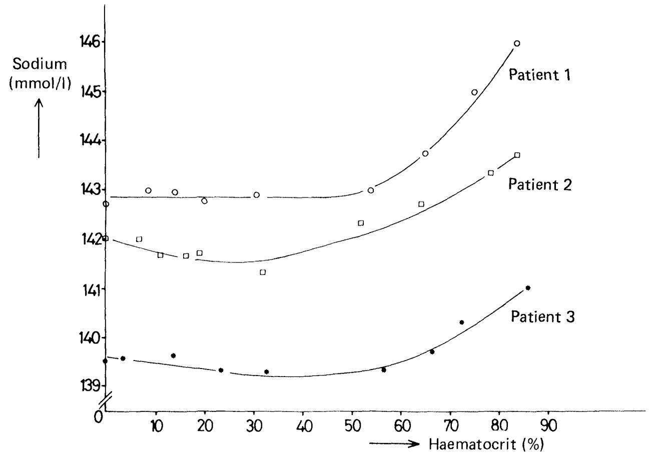

*Figure 2. Influence of haematocrit on the sodium concentration for three patients.*

### *Influence of electrode drift after a standby period*

As the electrode potential in the standby mode tends to drift away, a phenomenon also observed with other ISE analysers, the repeated measurement of a sample after a standby period gives displayed concentrations that tend to drift towards a constant lower value. As the sodium concentration is displayed in three digits, this phenomenon is best illustrated with the change of the potassium concentration. The difference between the first potassium measurements after standby periods and steady state concentrations is about 0·13 mmol/l.

### *Direct potentiometry versus flame photometry*

Figure 3 shows the comparison of sodium and potassium concentrations measured by direct potentiometry and flame photometry in serum samples from 50 patients. Flame photometric concentrations compared with Corning 902 concentrations were 99·1 ± 1·1% as great for sodium and 99·3 ± 3·3% as great for potassium. These differences were close to the percentages reported by Ladenson [6] (97·2 ± 2·1% for sodium and 98·1 ± 2·4% for potassium). The differences between direct potentiometry and flame photometry depend partly on the composition of the direct potentiometric standard solutions and therefore on the fact that ISEs respond to the ionic activity

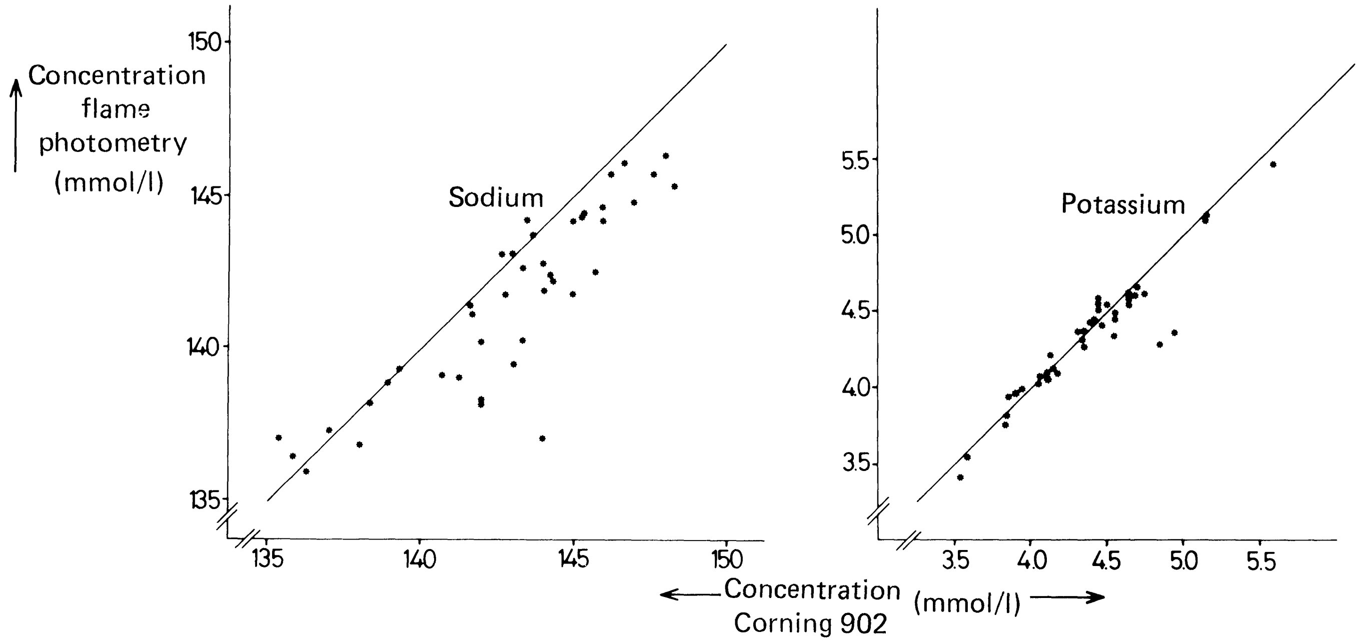

*Figure 3. Comparison of sodium and potassium concentrations analysed with direct potentiometry and flame photometry.*

(mmol/kg water) of an ion and not to the concentration (mmol/l serum). When a standard solution with the same matrix as the serum sample is used (so there is no residual liquid junction potential and the activity coefficients in both solutions are equal), a 7% higher concentration of serum samples measured by direct potentiometry than by flame photometry is found (based on the assumption that the average water content of serum is 93%). When standard solutions with dissimilar matrices are used as the serum samples, other direct potentiometry-flame photometry differences depending on the ionic environment, pH, buffer, additives etc. of the standard solution are found.

### General comments

### *Instrument use*

The Corning 902 sodium/potassium analyser is extremely simple to operate because it has just three different push buttons (cal, slope and measure) with LED indicators that can be off, on, or flashing. When all the indicators are off the instrument is in the ready mode and a calibration or sample measurement can be performed. When a LED indicator is on, then that specific function is in progress; a flashing LED indicates that that specific function has to be performed by pushing the cal button or by offering the slope standard and then pushing the slope button. The results continue to be displayed until a new sample is aspirated. Results which are unreliable due to unstable readings are displayed as flashing concentrations.

### *Instruction manual*

The instruction manual is very clear and contains all the necessary operating instructions, precautions and hazards and a maintenance chapter. It also includes a troubleshooting guide with a series of flow diagrams for the different displayed error codes.

### Acknowledgements

The authors wish to thank Corning Ltd and AHS Nederland B.V. for their co-operation in this study. Also Harrie Evers for technical assistance and Carien Witte for help in the preparation of the manuscript.

### References

1. Waugh, W. H., *Metabolism*, **8** (1969), 706.

2. Shyr, C. and Young, C. C., *Clinical Chemistry*, **10** (1980), 1517.

3. Forrest, A. R. W. and Shenkin, A., *Lancet* (6 December 1980), 1256.

4. Vader, H. L. and Vink, C. L. J., *Clinica Chimica Acta*, **65** (1975), 379.

5. Ladenson, J. H., *Clinical Chemistry*, **10** (1977), 1912.

6. Ladenson, J. H., *Clinical Chemistry*, **5** (1979), 757.

7. Annan, W., Kirwan, N. A., Robertson, W. S., Teasdale, P. R. and Ager, B. P., *Journal of Automatic Chemistry*, **2** (1980), 212.

8. Maas, A. H. J., *Clinica Chimica Acta*, **28** (1970), 373.

9. Bijster, P., Vader, H. L. and Vink, C. L. J., *Annals of Clinical Biochemistry* (in press).

